# Supplementary material for: Objective Structured Assessment of Debriefing (OSAD) in simulation-based medical education: Translation and validation of the German version
Source: PLoS One. 2020 Dec 31;15(12):e0244816. doi: 10.1371/journal.pone.0244816 (PMC7774931; doi:10.1371/journal.pone.0244816)
Supplement: S1 Appendix — (PDF) [file pone.0244816.s001.pdf]

Ein objektives strukturiertes Debriefingauswertungsinstrument (G-OSAD)

Eine deutsche Version des englischsprachigen, evidenzbasierten Instruments Objective Structured Assessment of Debriefing (OSAD) zur Erfassung der Qualität von Debriefings*

Autoren: S Abegglen[1], A Krieg[2], H Eigenmann[1], R Greif[2]

[1] Abteilung Gesundheitspsychologie und Verhaltensmedizin, Institut für Psychologie, Universität Bern, Schweiz

[2] Department of Anesthesiology and Pain Therapy, Bern University Hospital, University of Bern, Bern, Switzerland

* https://www.imperial.ac.uk/patient-safety-translational-research-centre/education/training-materials-for-use-in-research-and-clinical-practice/the-observational-structured/

Objektive strukturierte Auswertung von Debriefings (G-OSAD)					
	**1**	**2**	**3**	**4**	**5**
**1. Herangehensweise**	Konfrontative, wertende Herangehensweise		Versucht, eine gute Beziehung zu den Teilnehmenden herzustellen, ist aber zu kritisch oder zu informell in der Herangehensweise		Etabliert und erhält eine gute Beziehung zu den Teilnehmenden; ehrliche, aber nicht verletzende Herangehensweise, um eine psychologisch sichere Atmosphäre zu schaffen
**2. Etabliert Lernatmosphäre**	Erwartungen der Teilnehmenden bleiben unklar; keine Regeln für das Engagement der Teilnehmenden		Erklärt den Zweck des Debriefings oder der Simulation, klärt jedoch nicht die Erwartungen der Teilnehmenden		Erklärt den Zweck des Debriefings und klärt Erwartungen und Lernziele der Teilnehmenden zu Beginn
**3. Einbeziehen der Teilnehmenden**	Frontaler Unterricht der Instruktor*innen, ohne Einbezug der passiven Teilnehmenden		Teilnehmende werden meist durch geschlossene Fragen in die Diskussion miteinbezogen, passive Teilnehmende werden nicht aktiv dazu eingeladen, sich einzubringen		Die Teilnehmenden werden mittels offener Fragen dazu eingeladen, die Diskussion aktiv mitzugestalten
**4. Reaktions-Phase**	Keine Berücksichtigung der Reaktionen der Teilnehmenden oder der emotionalen Auswirkungen des Erlebten		Befragt die Teilnehmenden nach ihrem Empfinden, ergründet deren Reaktionen auf das Erlebte jedoch nicht umfassend		Ergründet die Reaktionen der Teilnehmenden auf das Erlebte umfassend und geht angemessen auf unzufriedene Teilnehmende ein
**5. Beschreibungs-Phase**	Keine Gelegenheit zur Selbstreflexion. Die Teilnehmenden werden nicht zur Beschreibung der tatsächlichen Ereignisse im Szenario aufgefordert.		Ungefähre Beschreibung von Ereignissen durch Instruktor*in, aber mit wenig Selbstreflexion der Teilnehmenden		Ermutigt die Teilnehmenden zur schrittweisen Selbstreflexion der Ereignisse des Szenarios
**6. Analyse-Phase**	Keine Exploration der Gründe und Konsequenzen der Handlungen mit den Teilnehmenden		Teilweise Exploration der Gründe und Konsequenzen der Handlungen durch Instruktor*in (ohne Beteiligung der Teilnehmenden); keine Gelegenheit, diese mit vorherigen Erfahrungen zu verknüpfen		Hilft den Teilnehmenden, die Gründe und Konsequenzen der Handlungen zu explorieren, indem konkrete Beispiele erörtert werden und mit vorherigen Erfahrungen verknüpft werden
**7. Diagnose-Phase**	Kein Feedback zu klinisch-technischen Fertigkeiten oder zur Zusammenarbeit im Team. Identifiziert Leistungslücken nicht oder bietet keine positive Verstärkung.		Feedback nur zu klinisch-technischen Fertigkeiten; Fokus auf Fehler und nicht nur auf potenziell veränderbare Verhaltensweisen		Gibt objektives Feedback zu klinisch-technischen Fertigkeiten und der Zusammenarbeit im Team; erkennt korrektes Verhalten wie auch Leistungslücken und fokussiert auf potenziell veränderbare Verhaltensweisen
**8. Anwendungs-Phase**	Keine Gelegenheit für die Teilnehmenden, Strategien für zukünftige Verbesserungen zu identifizieren oder zentrale Lernpunkte zu festigen		Teilweise Diskussion zentraler Lernpunkte und Verbesserungs-Strategien jedoch mangelnder Transfer in die zukünftige klinische Praxis		Verstärkt von Teilnehmenden identifizierte zentrale Lernpunkte und hebt hervor, wie Verbesserungs-Strategien in der zukünftigen klinischen Praxis angewandt werden können

Hinweise für die Anwendung von G-OSAD

- Sie beobachten und bewerten die Instruktoren in seiner Fähigkeit, ein Debriefing zu leiten (NICHT die Teilnehmenden).
- Bitte lesen Sie den gesamten Bewertungsbogen vor Beginn des Debriefings, um sicherzustellen, dass Sie die für die Bewertung relevanten Verhaltensweisen der Instruktoren beobachten.
- Sie beurteilen die Instruktoren in acht Kategorien (Definitionen siehe unten.) auf einer Skala von 1 (sehr schlecht) bis 5 (sehr gut).
- Um Ihnen die Beurteilung der beobachtbaren Verhaltensweisen zu erleichtern, sind Beispiele zur Vergabe von 1, 3 oder 5 Punkten angeführt (siehe unten). Wenn Sie sich für eine Bewertung dazwischen entscheiden, vergeben Sie 2 oder 4 Punkte.
- Definitionen und Beispiele einiger dieser Verhaltensweisen finden Sie untenstehend.
- Während des Simulationskurses sollte Kategorie 2 (Lernklima) am Anfang des ersten Debriefings beurteilt werden und daher ist es nicht angebracht, diese Kategorie bei weiteren Debriefings erneut zu bewerten.
- In Gruppen-Debriefings ist es wichtig, dass die Instruktoren alle Teilnehmenden einbezieht, um 5 Punkte zu erhalten. Da es Einzel- und Gruppen-Debriefings gibt, können sich die Beispiele auf „die Teilnehmenden" beziehen.
- Für die Verwendung von G-OSAD als Leitlinie dafür, was in einem Debriefing abgedeckt werden sollte, ist neben den vorliegenden G-OSAD-Materialien kein weiteres Training notwendig. Zur Nutzung von G-OSAD für die Qualitätsmessung von Debriefings wird ein vorgängiges Rater-Training empfohlen, um zuverlässige Resultate zu erzielen.

G-OSAD - Definitionen und Verhaltensbeispiele der Instruktoren

Kategorie	Definition	Beispiel für 1 Punkt	Beispiel für 5 Punkte
1. Herangehensweise	*Herangehensweise an das Debriefing; angemessene Begeisterung und positive Einstellung; zeigt Interesse gegenüber den Teilnehmenden, indem eine gute Beziehung erstellt und aufrechterhalten und das Debriefing positiv beendet wird*	"In diesem Szenario hast du eine Menge Fehler gemacht, was schlecht ist, da ich davon ausgehe, dass du dieses Szenario bereits einmal gesehen hast. "	"Lasst uns mit einer Vorstellungsrunde beginnen, sodass wir die Hintergründe und bisherigen Erfahrungen der anderen besser verstehen können."
2. Etabliert Lernatmosphäre	*Einführung in die Simulation durch Klärung der Erwartungen an die Teilnehmenden im Debriefing; Betonung von Grundregeln der Vertraulichkeit und des Respekts; Ermutigung der Teilnehmenden, eigene Lernziele zu formulieren*	"Mich interessiert nicht, welchen Zweck ihr in dieser Simulation seht, aber ich weiss, was ich euch beibringen will und das ist sehr wichtig für mich."	"Bitte beginnt, indem ihr erklärt, was ihr aus diesem Debriefing mitnehmen möchtet. Alles, was wir diskutieren, wird vertraulich behandelt."
3. Einbeziehen der Teilnehmenden	*Aktives Einbeziehen aller Teilnehmenden in die Diskussion, indem offene Fragen gestellt werden, um ihre Gedankengänge zu ergründen und Stille genutzt wird, um eigene Beiträge zu ermutigen. Den Teilnehmenden die grösste Redezeit zugestehen, damit tief und nicht nur oberflächlich gelernt wird*	"Ich werde euch nun beibringen, wie man es richtig macht und möchte, dass ihr alle still seid und mir zuhört."	"Kannst du uns als Teamleiter*in beschreiben, was an diesem Punkt des Szenarios vor sich ging? Warum, denkt ihr, ist dies passiert? "
4. Reaktions-Phase	*Ergründen welche emotionalen Auswirkungen das Szenario auf die Teilnehmenden hat*	"Ich verstehe nicht, wieso dich die Ereignisse des Szenarios so aufwühlen, das ist bisher noch bei niemandem vorgekommen."	"Dieser Teil des Szenarios wirkte auf uns Beobachter*innen sehr stressig, wie hast du dich dabei gefühlt? Hat dies den Rest des Szenarios beeinflusst und falls ja, wie?"
5. Beschreibungs-Phase	*Schrittweise, faktenbezogene Selbstreflexion der Vorkommnisse im Szenario; klären aller klinisch-technischen Fragen zu Beginn, um eine kontinuierliche Reflexion aller Teilnehmenden während der Analyse- und Anwendungsphase zu ermöglichen. Verknüpfung mit bisherigen Erfahrungen*	"Ich kann euch genau sagen, was und warum ihr es so gemacht habt."	"Könnt ihr von Anfang an Schritt für Schritt beschreiben, was ihr beobachtet habt, damit wir ein gemeinsames Verständnis der Ereignisse des Szenarios erlangen."
6. Analyse-Phase	*Ergründen der Denkprozesse, welche den Handlungen der Teilnehmenden zugrunde liegen, indem spezifische Beispiele beobachtbaren Verhaltens genutzt werden, damit sie die Ereignisse der Simulation nachvollziehen können*	"Es hat keinen Sinn, dich zu fragen, warum du das so gemacht hast, aber beim nächsten Mal musst du es anders machen."	"Was denkst du, weshalb geschah dies genau in jenem Moment? Was hat dich abgelenkt?"
7. Diagnose-Phase	*Den Teilnehmenden ermöglichen, ihre Leistungslücken und Verbesserungsstrategien zu identifizieren; wobei nur veränderbare Verhaltensweisen angegangen werden und somit objektives und strukturiertes Feedback gegeben wird*	"Das lief ganz okay, denke ich. Aber du hast nichts besonders gut gemacht."	"Du hast bemerkt, dass deinem Team nicht klar wurde, wie besorgt du warst. Kannst du dir vorstellen, wie du das nächste Mal deine Bedenken klarer ausdrücken könntest?"
8.Anwendungs-Phase	*Zusammenfassung der Lernpunkte und Verbesserungsstrategien, die durch die Teilnehmenden im Debriefing formuliert wurden, sowie ein Ausblick darüber, wie diese in der zukünftigen klinischen Praxis umgesetzt werden könnten*	"Also, machst du es beim nächsten Mal besser? Ich denke, du weisst selbst, was du im Szenario falsch gemacht hast. Lasst uns hier abschliessen."	"Kannst du die zentralen Lernpunkte zusammenfassen? Wie würdest du eine ähnliche Situation am Arbeitsplatz in Zukunft anders handhaben?"
