# Supplementary material for: Objective Structured Assessment of Debriefing (OSAD) in simulation-based medical education: Translation and validation of the German version
Source: PLoS One. 2020 Dec 31;15(12):e0244816. doi: 10.1371/journal.pone.0244816 (PMC7774931; doi:10.1371/journal.pone.0244816)
Supplement: S2 Appendix — (PDF) [file pone.0244816.s002.pdf]

Typical questionnaire answers about the German version of the OSAD

Question: What measures OSAD in your view?

Answers (Examples):

- The competence of the instructor and therefore the quality of the debriefing.
- The OSAD instrument measures the instructor's ability and competence to conduct a debriefing.
- Based on a clearly defined points system, the qualitative aspects of a debriefing are evaluated in terms of course technique, didactics, employee involvement and attitude of the debriefers. The aspects are broken down and analysed according to the known debriefing phases.

Question: How well can the instructor's ability to conduct a debriefing be assessed using the OSAD?

Extremely bad									Extremely good
□ 1	□ 2	□ 3	□ 4	□ 5	□ 6	□ 7	□ 8	□ 9	□10

Question: Which adjustments in the questions could improve this assessment?

Answers (Examples):

- I think it’s very comprehensive, I see no possibility for improvement.
- I think the instrument is very good and I can imagine it well in use.
- I find the definitions and examples easy to understand and exemplary. No wishes for additions/adaptations.
- In the case of category 5 reflection, I fear that this could be misunderstood in German. From a pedagogical point of view the whole debriefing is a reflection, at least the scene understands it as such a process. In my opinion, category 5 is more aimed at describing the events during the scenario.
- In my opinion, there are categories that need to be assessed over the entire duration of the debriefing (1-3) and others that focus essentially on a specific part of it (4-8). Since most debriefing models have a phase subdivision, it would, in my opinion, improve the applicability if this reference was made clearly visible. On the one hand

this should be obvious in the evaluation grid (partly this is implemented in your version), on the other hand this again speaks for an initial rater training.

Question: Are the instructions "how to apply" on page 3 of the G-OSAD instrument understandable?

☐ Yes. ☐ No. The following is not understandable or would have to be supplemented:

Answers (Examples):

- The instructions are in my opinion easy to understand.
- Here I would like to object a "yes, but". From my point of view there is no way around a rater training here. Before you use it for an assessment, the tool should be explained. Ideally, a team of debriefers should work out a common meaning of the individual points.

Question: Further comments or suggestions for improvement:

Answers (Examples):

- I am very excited about the application phase and consider it well thought out.
- I find the OSAD an interesting tool, which certainly contributes to the performance increase of the Instructor*in.
- I consider the examples for a 1 point rating - at least in part - to be very strikingly formulated. Perhaps you should reconsider.
- I think the points 1, 3 or 5 are quickly assigned, it is more difficult with the ratings 2 and 4. If necessary, make room for the reason (larger field), also define it or leave it out.
