# Supplementary material for: Objective Structured Assessment of Debriefing (OSAD) in simulation-based medical education: Translation and validation of the German version
Source: PLoS One. 2020 Dec 31;15(12):e0244816. doi: 10.1371/journal.pone.0244816 (PMC7774931; doi:10.1371/journal.pone.0244816)
Supplement: Attachment — Submitted filename: PONE-D-20-32979_reviewer_comments.pdf [file pone.0244816.s003.pdf]

PLOS ONE

Objective Structured Assessment of Debriefing (OSAD) in simulation-based medical education: translation and validation of the German version

--Manuscript Draft--

**Manuscript Number:**	PONE-D-20-32979
**Article Type:**	Research Article
**Full Title:**	Objective Structured Assessment of Debriefing (OSAD) in simulation-based medical education: translation and validation of the German version
**Short Title:**	German translation and validation of the OSAD
**Corresponding Author:**	Sandra Abegglen, Ph.D.<br>University of Bern<br>Bern, Bern SWITZERLAND
**Keywords:**	Objective Structured Assessment of Debriefing (OSAD); debriefing; quality; assessment; simulation-based medical education; translation; German
**Abstract:**	Debriefing is essential for effective learning during simulation-based medical education. To assess the quality of debriefings, reliable and validated tools are necessary. One widely used validated tool is the Objective Structured Assessment of Debriefing (OSAD), which was originally developed in English. The aim of this study was to translate the OSAD into German, and to evaluate the reliability and validity of this German version (G-OSAD) according the ‘ Standards of Educational and Psychological Measurement’ . In Phase 1, the validity evidence based on content was established by a multistage cross-cultural adaptation translation of the original English OSAD. Additionally, we collected expert input on the adequacy of the content of the G-OSAD to measure debriefing quality . In Phase 2, three trained raters assessed 57 video recorded debriefings to gather validity evidence based on internal structure. Interrater reliability, test-retest reliability, internal consistency, and composite reliability were examined. Finally, we assessed the internal structure by applying confirmatory factorial analysis. The expert input supported the adequacy of the content of the G-OSAD to measure debriefing quality. Interrater reliability (intraclass correlation coefficient) was excellent for the average ratings (three raters: ICC = 0.848; two raters: ICC = 0.790), and good for the single rater (ICC = 0.650). Test-retest reliability was excellent (ICC = 0.976), internal consistency was acceptable (Cronbach’s a = 0.865), and composite reliability was excellent (ω = 0.93) . Factor analyses supported the unidimensionality of the G-OSAD, which indicates that these G-OSAD ratings measure debriefing quality as intended. T he G-OSAD shows good psychometric qualities to assess debriefing quality, which are comparable to the original OSAD. Thus, this G-OSAD is a tool that has the potential to optimise the quality of debriefings in German-speaking countries.
**Order of Authors:**	Sandra Abegglen, Ph.D.<br>Andrea Krieg<br>Helen Eigenmann<br>Robert Greif
**Additional Information:**	
**Question**	**Response**
**Financial Disclosure**<br><br>Enter a financial disclosure statement that describes the sources of funding for the work included in this submission. Review the submission guidelines for detailed requirements. View published research articles from *PLOS ONE* for specific	The Department of Anaesthesiology and Pain Medicine, Bern University Hospital, Bern, Switzerland (http://www.anaesthesiologie.insel.ch/de/research/) granted a departmental research grant (GRRD-1-19) to RG. No other external funding was obtained. The funders had no role in study design, data collection and analysis, decision to publish, or preparation of the manuscript.

Powered by Editorial Manager® and ProduXion Manager® from Aries Systems Corporation

examples.<br><br>This statement is required for submission and **will appear in the published article** if the submission is accepted. Please make sure it is accurate.<br><br>**Unfunded studies**<br>Enter: *The author(s) received no specific funding for this work.*<br><br>**Funded studies**<br>Enter a statement with the following details:<br>• Initials of the authors who received each award<br>• Grant numbers awarded to each author<br>• The full name of each funder<br>• URL of each funder website<br>• Did the sponsors or funders play any role in the study design, data collection and analysis, decision to publish, or preparation of the manuscript?<br>• **NO** - Include this sentence at the end of your statement: *The funders had no role in study design, data collection and analysis, decision to publish, or preparation of the manuscript.*<br>• **YES** - Specify the role(s) played.<br><br>* typeset	
**Competing Interests**<br><br>Use the instructions below to enter a competing interest statement for this submission. On behalf of all authors, disclose any competing interests that could be perceived to bias this work—acknowledging all financial support and any other relevant financial or non-financial competing interests.<br><br>This statement **will appear in the published article** if the submission is accepted. Please make sure it is accurate. View published research articles from *PLOS ONE* for specific examples.	The authors have declared that no competing interests exist.

**NO authors have competing interests**<br><br>Enter: *The authors have declared that no competing interests exist.*<br><br>**Authors with competing interests**<br><br>Enter competing interest details beginning with this statement:<br><br>*I have read the journal's policy and the authors of this manuscript have the following competing interests: [insert competing interests here]*<br><br>* typeset	
**Ethics Statement**<br><br>Enter an ethics statement for this submission. This statement is required if the study involved:<br><br>• Human participants<br>• Human specimens or tissue<br>• Vertebrate animals or cephalopods<br>• Vertebrate embryos or tissues<br>• Field research<br><br>Write "N/A" if the submission does not require an ethics statement.<br><br>General guidance is provided below. Consult the submission guidelines for detailed instructions. **Make sure that all information entered here is included in the Methods section of the manuscript.**	The Bern Cantonal Ethics Committee (KEK-Bern) waived the need for ethics approval according to the Swiss Act on Human Research.

**Format for specific study types**<br><br>**Human Subject Research (involving human participants and/or tissue)**<br>• Give the name of the institutional review board or ethics committee that approved the study<br>• Include the approval number and/or a statement indicating approval of this research<br>• Indicate the form of consent obtained (written/oral) or the reason that consent was not obtained (e.g. the data were analyzed anonymously)<br><br>**Animal Research (involving vertebrate animals, embryos or tissues)**<br>• Provide the name of the Institutional Animal Care and Use Committee (IACUC) or other relevant ethics board that reviewed the study protocol, and indicate whether they approved this research or granted a formal waiver of ethical approval<br>• Include an approval number if one was obtained<br>• If the study involved *non-human primates*, add *additional details* about animal welfare and steps taken to ameliorate suffering<br>• If anesthesia, euthanasia, or any kind of animal sacrifice is part of the study, include briefly which substances and/or methods were applied<br><br>**Field Research**<br><br>Include the following details if this study involves the collection of plant, animal, or other materials from a natural setting:<br>• Field permit number<br>• Name of the institution or relevant body that granted permission	
**Data Availability**<br><br>Authors are required to make all data underlying the findings described fully available, without restriction, and from the time of publication. PLOS allows rare exceptions to address legal and ethical concerns. See the PLOS Data Policy and FAQ for detailed information.	Yes - all data are fully available without restriction

Powered by Editorial Manager® and ProduXion Manager® from Aries Systems Corporation

A Data Availability Statement describing where the data can be found is required at submission. Your answers to this question constitute the Data Availability Statement and **will be published in the article**, if accepted.<br><br>**Important:** Stating 'data available on request from the author' is not sufficient. If your data are only available upon request, select 'No' for the first question and explain your exceptional situation in the text box.<br><br>Do the authors confirm that all data underlying the findings described in their manuscript are fully available without restriction?	
**Describe where the data may be found in full sentences. If you are copying our sample text, replace any instances of XXX with the appropriate details.**<br><br>• If the data are **held or will be held in a public repository**, include URLs, accession numbers or DOIs. If this information will only be available after acceptance, indicate this by ticking the box below. For example: *All XXX files are available from the XXX database (accession number(s) XXX, XXX.*).<br>• If the data are all contained **within the manuscript and/or Supporting Information files**, enter the following: *All relevant data are within the manuscript and its Supporting Information files.*<br>• If neither of these applies but you are able to provide **details of access elsewhere**, with or without limitations, please do so. For example:<br><br>*Data cannot be shared publicly because of [XXX]. Data are available from the XXX Institutional Data Access / Ethics Committee (contact via XXX) for researchers who meet the criteria for access to confidential data.*<br><br>*The data underlying the results presented in the study are available from (include the name of the third party*	All datasets are available from the figshare database (doi: 10.6084/m9.figshare.13061555).

and contact information or URL).<br>• This text is appropriate if the data are owned by a third party and authors do not have permission to share the data.<br><br>* typeset	
Additional data availability information:	Tick here if the URLs/accession numbers/DOIs will be available only after acceptance of the manuscript for publication so that we can ensure their inclusion before publication.

Powered by Editorial Manager® and ProduXion Manager® from Aries Systems Corporation

Click here to access/download;Manuscript;manuscript_German version of

Objective Structured Assessment of Debriefing (OSAD) in simulation-based medical education: translation and validation of the German version

Sandra Abegglen[a,*], Andrea Krieg[b], Helen Eigenmann[a], Robert Greif[b,c]

[a]Department of Health Psychology and Behavioural Medicine, Institute of Psychology, University of Bern, Bern, Switzerland

[b]Department of Anaesthesiology and Pain Therapy, Bern University Hospital, University of Bern, Bern, Switzerland

[c]School of Medicine, Sigmund Freud University Vienna, Vienna, Austria

***Corresponding author**

E-mail: sandra.abegglen@psy.unibe.ch (SA)

Abstract

Debriefing is essential for effective learning during simulation-based medical education. To assess the quality of debriefings, reliable and validated tools are necessary. One widely used validated tool is the Objective Structured Assessment of Debriefing (OSAD), which was originally developed in English. The aim of this study was to translate the OSAD into German, and to evaluate the reliability and validity of this German version (G-OSAD) according the 'Standards of Educational and Psychological Measurement'. In Phase 1, the validity evidence based on content was established by a multistage cross-cultural adaptation translation of the original English OSAD. Additionally, we collected expert input on the adequacy of the content of the G-OSAD to measure debriefing quality. In Phase 2, three trained raters assessed 57 video recorded debriefings to gather validity evidence based on internal structure. Interrater reliability, test-retest reliability, internal consistency, and composite reliability were examined. Finally, we assessed the internal structure by applying confirmatory factorial analysis. The expert input supported the adequacy of the content of the G-OSAD to measure debriefing quality. Interrater reliability (intraclass correlation coefficient) was excellent for the average ratings (three raters: ICC = 0.848; two raters: ICC = 0.790), and good for the single rater (ICC = 0.650). Test-retest reliability was excellent (ICC = 0.976), internal consistency was acceptable (Cronbach's $\alpha = 0.865$), and composite reliability was excellent ($\omega = 0.93$). Factor analyses supported the unidimensionality of the G-OSAD, which indicates that these G-OSAD ratings measure debriefing quality as intended. The G-OSAD shows good psychometric qualities to assess debriefing quality, which are comparable to the original OSAD. Thus, this G-OSAD is a tool that has the potential to optimise the quality of debriefings in German-speaking countries.

Introduction

Simulation-based medical education (SBME) has gained increasing importance for learning of patient care and safety in healthcare over the last two decades [1, 2]. Research supports its general effectiveness for the improvement of learner knowledge [2], skills enhancement [2,3], and behavioural changes in the clinical settings [2,4], as well as for patient-related outcomes [2,2]. The subsequent debriefing is an integral component of learning by simulation [5,6,7].

This debriefing is defined as a guided discussion in which two or more people purposefully interact with each other to reflect and analyse their emotional states, thoughts and actions during and after the simulations. An effective debriefing provides insights from the experience, and allows application of the lessons learned to future medical practice, to improve performance [6,8,9]. Experiential learning recognizes that the active role of the participants during this reflection of their experience to generate new knowledge is important for the learning process itself. The instructor guides this process and supports the participants in making sense of the events they have experienced [5,10,11]. The facilitation of high-quality debriefing to maximise this learning has been identified as a challenge, and indeed an art, that requires continuous education [5,6,10,11].

Debriefing improves medical knowledge and technical and non-technical skills [12,13], and can enhance performances by ~25% [14]. In contrast, SBME without debriefing offers lesser benefit to the participants [15,16]. Despite the essential role of debriefing for learning in SBME, more research on the quality and efficacy of debriefing has been claimed to optimise the efficacy of the debriefing [14,14]. Tools to assess the quality of debriefing have been developed [17,18]. One of these tools is the Objective Structured Assessment of Debriefing (OSAD), which was developed in English in the UK, based on evidence and end-user opinion [17]. The OSAD comprises eight categories of effective high-quality debriefing, which are

anchored to a behavioural rating scale. The OSAD serves several purposes: (1) to assess the quality of debriefing; (2) to provide feedback to instructors; (3) to enable relevant research; (4) to guide novice instructors and clinical trainers; (5) to exchange best practices; and (6) to promote high standards in debriefing [17,19].

No German version of the OSAD has been devised for simulation research and debriefing in SBME. Therefore, this study translated the original English version of the OSAD into German and analysed its psychometric properties in German, to create the first validated German version of the OSAD (G-OSAD).

Methods

The eight categories of high-quality debriefing of the original English OSAD [17] include approach of the facilitator, establishment of a learning environment, learner engagement, gauging learner reaction, descriptive reflection, analysis of performance, diagnosis of performance gaps, and application to future clinical practice. A 5-point scale (1, minimum; 5, maximum) was used to rate the performance of the instructor for each category, which resulted in a global score from 8 (minimum) to 40 (maximum). The supporting evidence for its use has been provided through simulations and testing in clinical practice, and includes the face, content and concurrent validity, the interrater reliability, the test-retest validity, and the internal consistency [17,19].

We used the framework of validity described in the ‘Standards of Educational and Psychological Measurement’ [20] and collected validity evidence elements based on the ‘content’ and the ‘internal structure’ in two phases. In Phase 1, validity evidence was gathered based on the content by translation, back-translation and cross-cultural adaptation, to ensure consistency between the original English OSAD and the G-OSAD developed here. Additionally, we obtained expert input on whether the content of the G-OSAD was an adequate

representation of the construct quality of debriefing. In Phase 2, video recorded debriefings of SBME were rated using the original English OSAD and the G-OSAD, to collect validity evidence based on the internal structure and to compare the ratings of the original English OSAD and the G-OSAD developed.

Phase 1: Validity evidence based on content

A multi-stage process based on the translation, review, adjudication, pre-testing and documentation (TRAPD) methodology [21] guided the translation and cross-cultural adaptation process, with each stage documented. This was based on the following processes:

(1) Three psychologists, three medical practitioners and three experienced simulation instructors each individually translated the original English OSAD into German.

(2) Each of these professional groups then agreed upon a consensus version.

(3) One person in each of the professional groups (authors AK, RG, HE) finally agreed on a German version in a consensus meeting.

(4) Two bilingual speakers with medical backgrounds (German/English native: MB, FU [Acknowledgements]) independently back-translated this German version to English for a consensus back-translation. Both were blinded to the original English OSAD.

(5) An expert committee consisting of two psychologists (authors SA, HE), one physician (author AK) and one native English speaker (MB, Acknowledgements) compared the consensus back-translation to the original OSAD. Where there were differences, the German version was adapted to match the meaning of the original English OSAD.

(6) This German version, together with a semi-structured questionnaire, was sent to nine expert instructors of SBME (3 physicians, 3 nurses, 2 paramedics, 1 midwife; working experience: 11-39 years). The semi-structured questionnaire included open questions regarding the view of the participants, the comprehensibility of the instructions for use, further suggestions for

improvement, and the question: “How well can an instructor’s ability to conduct a debriefing be assessed by the German OSAD version?” (Likert-scale: 1, extremely poor; 10, extremely well).

(7) The expert committee (see point 5) then integrated the results from the semi-structured questionnaire into the final version of the G-OSAD (S1 Appendix).

This translation and the expert input constituted the validity evidence elements based on content.

Phase 2: Validity evidence based on internal structure

With written informed consent, 57 debriefings (duration, 20-59 min.) were video recorded to apply the G-OSAD. These debriefings were from SBME courses for anaesthesia residents and nurses at the Bern University Hospital Simulation Centre (Bern, Switzerland). Each debriefing was co-led by two instructors, who were previously trained according to EuSim-Group educational standards [22] (experienced simulation instructor trainers who provide simulation instructor courses in collaboration with medical and research partners). The instructor demographics were recorded.

Three psychology students received rater training, that comprised: (a) study of and discussions around the G-OSAD; (b) discussions of potential observer bias; (c) definition of technical and non-technical medical terms; (d) pilot rating of six video recorded debriefings, followed by discussions to establish common understanding of the rating categories. These newly trained raters evaluated the overall quality of debriefings conducted by the two simulation instructors by first watching the video recorded debriefings and taking notes, and then filling in the G-OSAD. After the rater training, each of them rated all of the 57 video recorded debriefings in a random order. Two months later, the same three raters scored six debriefings again, randomly selected from the original 57 (i.e., ~10% of the original sample),

to establish the test-retest reliability. Additionally, three different psychology students rated the same 57 debriefings using the original English OSAD, to compare these with the G-OSAD ratings (datasets available from [23]).

The raters were not able to score the second category of ‘establishes learning environment’. The explaining of the purpose of the debriefing and clarifying the objectives and expectations of the participants was held at the beginning of each simulation, and therefore were not part of the debriefings; therefore, this category was not video recorded and could not be analysed.

Statistical analysis

Statistical significance was set at $p < 0.05$ for all analyses. The mean scores and standard deviations were calculated for the Likert-scale questions of the semi-structured questionnaire on the content of the G-OSAD.

The intraclass correlation coefficients (ICCs; two-way random model, absolute agreement type) were calculated as the measures for interrater reliability for the total G-OSAD and for each category (except ‘establishes learning environment’; see above). The ICCs of both the single measures (ratings of one rater) and the average measures (means of the raters) were computed for comparisons of their reliability, and thereby to derive possible implications for the use of the G-OSAD.

The ICC (consistency type) of the average total G-OSAD ratings with the average total original English OSAD ratings was calculated as the measure of agreement between the ratings of the two versions. To estimate test-retest reliability, the ICC (absolute agreement type) was calculated based on the average total scores of the randomly selected 10% G-OSAD ratings.

Exploratory factor analysis was performed, followed by confirmatory factor analysis (CFA), based on the average ratings of the G-OSAD categories (excluding ‘establishes learning

environment'; see above). The adequacy of the observed data was evaluated using the Kaiser–Meyer–Olkin criterion and Bartlett's test of sphericity. The data analysis was conducted using the R-packages *lavaan* [24], *psych* [25] and *simsem* [26], in the R statistical language [27]. Preliminary analyses indicated violation of the assumption of multivariate normality of the G-OSAD items (Mardia's coefficient of skewness = 153.58; p <0.001). Thus, maximum likelihood estimation was used with robust standard errors and a Satorra–Bentler corrected test statistic [28]. As the χ^2 test is sensitive to sample size and complexity of the model [29], additional fit measures are reported for evaluation of the model fit. Based on recommendations [29], to define an adequate fit, the standardised root mean square residual and root mean square error of approximation should be <0.05, and the normed fit index, comparative fit index and Tucker–Lewis index should be >0.95. We performed bootstrapping [30] and applied Monte-Carlo re-sampling techniques to test the appropriateness of the model parameters, standard errors, confidence intervals and fit indices [30,31], because the sample size was only 57 debriefings. Finally, internal consistency was evaluated using Cronbach's alpha (α), and composite reliability using Omega (ω) [32].

Results

Phase 1: Validity evidence based on content

The G-OSAD was developed based on the original English OSAD, through translation, back-translation and cross-cultural adaptation, to match the content of the original version. Nine expert instructors of SBME answered the semi-structured questionnaire to provide evidence for the validity based on content. One instructor did not answer the question "How well can an instructor's ability to conduct a debriefing be assessed by the German OSAD version?". The mean score of the eight instructors was 8.9 ±1.0 on the 10-point Likert-scale. The open questions revealed that this G-OSAD is useful to assess the quality of debriefing, and has the

potential to contribute to performance improvement (see S2 Appendix for examples of typical answers). Suggestions from the instructors that deviated from the original English OSAD were not considered, to [illegible] deviate too much from the original English OSAD. This final G-OSAD then entered Phase 2.

Phase 2: Validity evidence based on internal structure

The 14 instructors who facilitated the 57 debriefings that were used to rate the G-OSAD were on average 43.3 ±8.8 years old, with mean working experience as instructors of 4.9 ±4.2 years; three were women (21%).

The descriptive statistics of the G-OSAD ratings of the three trained raters across all of the debriefings are summarised in Table 1.

Table 1. Descriptive statistics of the G-OSAD ratings of the three trained raters across all of the debriefings (N = 57).

**G-OSAD category**		**Rater 1**	**Rater 2**	**Rater 3**	**Mean**
1	Approach	3.72 ±0.67	3.74 ±0.52	3.75 ±0.51	3.74 ±0.51
2	Establishes learning environment[a]	-	-	-	-
3	Engagement of learners	4.48 ±0.82	4.07 ±0.82	4.18 ±0.85	4.24 ±0.73
4	Reaction phase	2.75 ±0.83	2.39 ±0.84	2.65 ±0.92	2.60 ±0.64
5	Description phase	4.61 ±0.82	3.95 ±0.77	4.04 ±0.76	4.05 ±0.65
6	Analysis phase	4.50 ±0.68	4.19 ±0.85	4.16 ±0.73	4.28 ±0.63
7	Diagnosis phase	4.63 ±0.67	4.46 ±0.60	4.40 ±0.73	4.50 ±0.56
8	Application phase	3.79 ±0.67	3.67 ±0.69	3.60 ±0.68	3.68 ±0.49
**9**	**Total score**	**28.02 ±3.76**	**26.46 ±3.32**	**26.77 ±3.58**	**27.08 ±3.16**

For understanding here, the categories of the G-OSAD are presented in English

[a], Category not assessed because it was not part of the debriefing videorecordings, and is therefore excluded

The interrater reliability (ICC) of the G-OSAD scores for each category (excluding ‘establishes learning environment’; see above) are reported in Table 2. The ICC for the average ratings of the three raters was 0.848 for the total score and for the different categories ranged between 0.531 to 0.863. The ICC for the average ratings of two raters was 0.790 for the total score and ranged between 0.429 to 0.812 for the categories. The single ratings yielded lower ICCs with 0.650 for the total score and a range of 0.274 to 0.678 for the categories (Table 2). ICC values <0.40 are considered poor, 0.40 to 0.59 fair, 0.60 to 0.74 good, and 0.75 to 1.00 excellent [33].

Table 2. Interrater reliability (as intraclass correlation coefficient) for the single and average ratings for each category across all of the debriefings (N = 57).

**G-OSAD category**		**Intraclass correlation coefficient [mean (95% confidence interval)]**		
		**Single rater****	**Two raters[b,*/**]**	**Three raters****
1	Approach	0.678 (0.552–0.782)	0.812 (0.680–0.889)	0.863 (0.787–0.915)
2	Establishes learning environment[a]	-	-	-
3	Engagement of learners	0.624 (0.469–0.749)	0.768 (0.538–0.872)	0.833 (0.726–0.899)
4	Reaction phase	0.318 (0.158–0.485)	0.479 (0.131–0.689)*	0.583 (0.360–0.739)
5	Description phase	0.534 (0.384–0.671)	0.692 (0.477–0.818)	0.775 (0.652–0.860)
6	Analysis phase	0.516 (0.358–0.659)	0.679 (0.436–0.815)	0.762 (0.626–0.853)
7	Diagnosis phase	0.540 (0.390–0.676)	0.697 (0.483–0.822)	0.779 (0.657–0.862)
8	Application phase	0.274 (0.112–0.446)	0.429 (-0.077–0.663)*	0.531 (0.275–0.707)
**9**	**Total score**	**0.650 (0.503–0.767)**	**0.790 (0.604–0.883)**	**0.848 (0.752–0.908)**

For understanding here, the categories of the G-OSAD are presented in English

[a], Category not assessed because it was not part of the debriefing videorecordings, and is therefore excluded

[b], Calculated as means of the three different possible pairs of raters.

*/**, p <0.001 for all except: *, p <0.05).

The test-retest reliability of the total average scores of the 10% G-OSAD ratings reassessed after 2 months showed ICC of 0.976 (95% confidence interval, 0.837–0.997; p = 0.001). These psychometrical characteristics are given in Table 3. The agreement between the average total G-OSAD ratings and the average total original English OSAD ratings was ICC = 0.586 (95% confidence interval, 0.297–0.756; p = 0.001).

Table 3. Psychometric properties of the original English Objective Structured Assessment of Debriefing (OSAD) [17] and the German OSAD.

**Psychometric property**	**Objective Structured Assessment of Debriefing**				
	**Original**	**German**			
	**English[a]**	**Overall**	**Single rater**	**Two raters**	**Three raters**
Cronbach's α	0.89	0.87 (CI 0.81–0.92)	--	--	--
Omega (ω)	n.a.	0.93	--	--	--
Test-retest reliability	0.89	0.98	--	--	--
**Interrater reliability** (ICC)					
Total score	0.88		0.65	0.79	0.87
Range (individual categories)	n.a.		0.27–0.68	0.43–0.81	0.53–0.86

ICC, intraclass correlation coefficient; CI, confidence interval

[a], ICC of the total score only reported; single and average ratings and Omega ω not reported

n.a., data not available

For the CFA based on the average ratings of the G-OSAD categories (excluding 'establishes learning environment'; see above) the adequacy of the observed data was confirmed by the Kaiser–Meyer–Olkin criterion of 0.83, and the Bartlett's test of sphericity of χ^2 (21) of 263.61 (p <0.001).

Exploratory factor analysis was performed to explore the dimensional structure of the G-OSAD. The results of the parallel analysis [34], the 'very simple structure criterion' [35], the inspection of the scree plot, and Velicer's minimum average partial test [36] indicated

extraction of one factor. CFA was also caried out to establish the factorial validity for the measurement model. The robust χ^2 test remained significant (χ^2[14, N = 57] = 31.08; p = 0.005). The fit indices for the overall fitting for the different one factor models are given in Table 4, which are acceptable according to the published guidelines [29]. Modification indices were examined to determine the areas of localised strain. No large modification indices (>10) were seen, and no *post-hoc* modifications were made to improve the overall model fit.

Table 4. Fitting statistics for the one factor model for all of the debriefings (N = 57).

One factor model	Absolute fit indices				Comparative fit indices		
	$\chi^2(df)$	χ^2/df	SRMR	RMSEA (90% CI)	NFI	CFI	TLI
Robust	31.075 (14)	2.22	0.086	0.146 (0.078–0.214)	0.87	0.92	0.88
Bootstrap	15.24 (14)	1.09	0.049	0.038 (0.00–0.179)	0.93	0.98	0.99
Monte Carlo simulation	15.68 (14)	--	0.066	0.043 (0.039–0.047)	--	0.96	0.97

Df, degrees of freedom; SRMR, standardised root mean square residual; RMSEA, root mean square error of approximation; NFI, normed fit index; CFI, comparative fit index; TLI, Tucker–Lewis index

Fitting of the internal structure

The standardised parameter estimates range was β = 0.20–0.94 (see Fig 1). Thus, in the final model, two categories (i.e., ‘reaction phase’, β = 0.200; ‘application phase’, β = 0.385) were little correlated with the latent structure. The full model is as shown in Fig 1.

Fig 1. **Factor structure of the G-OSAD.** Full model and standardised parameter estimates. Rectangles, observed variables; oval, latent construct. The paths from the latent construct to the observed variables indicate the standardised loading (β) of each variable. The arrows to the observed variables (left) indicate the measurement errors (ε). The category of ‘establishes learning environment’ was not assessed because it was not part of the video recordings of the debriefings, and therefore it was excluded.

Convergent validity

The average variance extracted is an indicator of the convergent validity, and thus it defines the variance captured by the construct in relation to the variance due to measurement error [37]. The average variance extracted for the global factor was 0.52, which exceeded the suggested minimum of 0.5 [37].

Item analysis and scale reliability

The item discrimination *(rjt),* internal consistency (Cronbach's α) and composite reliability (ω) of the categories of the G-OSAD were determined. The discrimination between participants of the categories with high *versus* low scores was satisfactory, with item discrimination coefficients from *rjt* = 0.60 to *rjt* = 0.88, except for the 'reaction phase' category (*rjt* = 0.44). Table 3 includes the data for internal consistency, given by Cronbach's α, and composite reliability, given by total ω.

Discussion

This study establishes a German version of the original English OSAD [17] according to the gathered validity evidence for the assessment of the quality of the debriefing. We used the framework of validity described in the 'Standards of Educational and Psychological Measurement' [20]. The evidence based on the content and internal structure supports the G-OSAD validly for the assessment of the quality of the debriefing.

According to the framework of validity [20], the thorough multistage translation, back-translation and cross-cultural adaptation serves as a first content validity element to ensure consistency with the evidence-based original English OSAD [17]. The supporting quantitative and qualitative feedback from expert instructors of SBME regarding the adequacy of the G-

OSAD content to assess the quality of the debriefings added further validity evidence based on content. In addition to the supporting validity evidence based on the content of the G-OSAD, that was gathered in the first phase, validity evidence elements based on the internal structure were collected in the second phase.

According to Cicchetti [33], the interrater reliability of the average ratings of the three raters was excellent for the total ICC score of 0.848, and for nearly all of the categories (except for ‘reaction phase’, ‘application phase’; Table 2). The ICCs of the average ratings of two raters were good to excellent (except for ‘reaction phase’, ‘application phase’). In contrast, the ratings of the individual raters were fair to good (again, except for ‘reaction phase’, ‘application phase’). This indicates that the average ratings of two raters are highly reliable for the total score for the measurement of the overall debriefing quality. For the differentiated analysis of the categories of G-OSAD, average ratings of three raters are needed to obtain highly reliable data. One open question is whether more extensive rater training might achieve highly reliable results with fewer raters.

Interestingly, the two categories of ‘reaction phase’ and ‘application phase’ yielded the lowest interrater reliabilities. Unfortunately, the report of the original OSAD [17] did not include the interrater reliability for the individual categories, only for the total score. Therefore, it is not possible to know whether the low interrater reliability of these two categories was a general issue also in the original OSAD, or whether it is due to the translation into German.

Test-retest reliability of the total scores (ICC = 0.98) was even higher than the reported value for the original OSAD scores (ICC = 0.89) [17] (Table 3). We do not know whether this difference can be attributed to the different retest periods (English OSAD, 6 month; G-OSAD, 2 months,). Regrettably, the other comparable debriefing quality scoring tool, the Debriefing Assessment for Simulation in Healthcare [18] does not report any test-retest reliability. This opens a wide field for future research.

The agreement between the G-OSAD ratings and the original English OSAD ratings can be considered as fair [33]. We consider that the ratings of the debriefing quality by the G-OSAD and the original English OSAD are comparable, which further supports the successful translation.

The OSAD was developed to assess the quality of debriefing. The quality of debriefing is a construct, and CFA can be used to explore the underlying structure of the OSAD ratings. For the original English OSAD, CFA is not available, but we conducted CFA to determine the underlying structure of the ratings of the G-OSAD categories. The CFA based on the ratings of the 57 debriefings indicated one u[illegible]rlying factor. As all of the categories of the G-OSAD included in the analysis could therefore be combined for this one underlying factor (Fig 1), this supports the interpretation that the construct quality of the debriefings is measured by the G-OSAD ratings.

In contrast to the other categories, the scoring of the 'reaction phase' and 'application phase' were not well covered by the factor (Fig 1). As reported in Table 2, these two categories had the lowest interrater reliabilities. This might be the reason for the low explanation through the factor, and does not necessarily indicate that they relate less to the factor.

The internal consistency of the G-OSAD scoring as assessed by Cronbach's α was acceptable (0.87) according to guidelines [38], and comparable to that for the original OSAD (0.89) (Table 3). This means that the raters scored the different categories of the G-OSAD similarly, which indicates that the categories are interrelated. Omega (ω) is seen as a more appropriate index of the extent to which all of the items in a scale measure the same latent variable [32]. The G-OSAD showed an excellent ω of 0.93, which supports that the ratings of the G-OSAD categories measure the same construct [32].

The three psychometric characteristics of the G-OSAD ratings of interrater reliability, test-retest reliability and internal consistency are comparable to those of the original English

OSAD (Table 3). This indicates that the thorough translation process successfully maintained its consistency with the original OSAD.

The validity evidence based on content and internal structure provide support that the G-OSAD ratings assess the construct of the debriefing quality. Thus, the G-OSAD is a tool that is now available for German-speaking areas that can be used to enhance debriefing quality.. As debriefings are an essential mechanism for learning in SBME [12,13,14,15,16], the G-OSAD might contribute to the effectiveness of SBME, which has been indicated as a concern in research in this field [2,4].

A study limitation here might be related to the generalisability if the translation process took place in one German-speaking area. However, here, German speakers from Austria, Germany and Switzerland translated the original English OSAD into German. Furthermore, this study did not include any validity evidence based on relations of the G-OSAD ratings to external criteria. Assessment of learning outcomes triggered by different levels of debriefing quality was beyond the scope of this research project. However, the original OSAD investigated relations to the participant evaluations of satisfaction and educational value, which have already been reported [17]. Another limitation is that the second category ('establishes learning environment') could not be assessed here, and was therefore not included in this analysis. As the establishment of a conducive learning environment is part of the briefing at the beginning of simulation courses [5], this was not part of the debriefing video recordings that were assessed here.

Conclusion

This study provides validity evidence based on content and internal structure for the valid assessment of the quality of debriefing by the G-OSAD. Therefore, the G-OSAD is a relevant tool to assess debriefing quality in German-speaking SBME.

Acknowledgements

The authors thank Renée Gastring, Gaelle Haas and Nadja Hornburg for their help with the data collection, and Yves Balmer, Mey Boukenna, Gianluca Comazzi, Tatjana Dill, Kai Kranz, Sibylle Niggeler and Francis Ulmer for their support in the translation process. They also thank all of the instructors for their feedback on the G-OSAD. Additionally, they thank Chris Berrie for critical review of the manuscript language.

References

1 Ziv A, Wolpe PR, Small SD, Glick S. Simulation-based medical education: an ethical imperative. Simul Healthc. 2006;1: 252-256. doi: 10.1097/01.SIH.0000242724.08501.63

2 Cook DA, Hatala R, Brydges R, Zendejas B, Szostek JH, Wang AT, et al. Technology-enhanced simulation for health professions education: a systematic review and meta-analysis. JAMA. 2011;306: 978-988. doi: 10.1001/jama.2011.1234

3 McGaghie WC, Issenberg SB, Cohen MER, Barsuk JH, Wayne DB. Does simulation-based medical education with deliberate practice yield better results than traditional clinical education? A meta-analytic comparative review of the evidence. Acad Med. 2011;86: 706-711. doi: 10.1097/ACM.0b013e318217e119

4 Boet S, Bould MD, Fung L, Qosa H, Perrier L, Tavares W, et al. Transfer of learning and patient outcome in simulated crisis resource management: a systematic review. Can J Anaesth. 2014;61: 571-582. doi: 10.1007/s12630-014-0143-8

5 Fanning RM, Gaba DM. The role of debriefing in simulation-based learning. Simul Healthc. 2007;2: 115-125. doi: 10.1097/SIH.0b013e3180315539

6 Dieckmann P, Molin Friis S, Lippert A, Østergaard D. The art and science of debriefing in simulation: ideal and practice. Med Teach. 2009;31: 287-294. doi: 10.1080/01421590902866218

7 McGaghie WC, Issenberg SB, Petrusa ER, Scalese RJ. A critical review of simulation-based medical education research: 2003–2009. Med Educ. 2010;44: 50-63. doi: 10.1111/j.1365-2923.2009.03547.x

8 Cheng A, Eppich W, Grant V, Sherbino J, Zendejas B, Cook DA. Debriefing for technology- enhanced simulation: a systematic review and meta- analysis. Med Educ. 2014;48: 657-666. doi: 10.1111/medu.12432

9 Rudolph JW, Simon R, Raemer DB, Eppich WJ. Debriefing as formative assessment: closing performance gaps in medical education. Acad Emerg Med. 2008;15: 1010-1016. doi: 10.1111/j.1553-2712.2008.00248.x

10 Dismukes RK, Gaba DM, Howard SK. So many roads: facilitated debriefing in healthcare. Simul Healthc. 2006;1: 23-25. doi: 10.1097/01266021-200600110-00001

11 Rudolph JW, Simon R, Dufresne RL, Raemer DB. There's no such thing as “nonjudgmental” debriefing: a theory and method for debriefing with good judgment. Simul Healthc. 2006;1: 49-55. doi: 10.1097/01266021-200600110-00006

12 Cheng A, Hunt EA, Donoghue A, Nelson-McMillan K, Nishisaki A, LeFlore J, et al. Examining pediatric resuscitation education using simulation and scripted debriefing: a multicenter randomized trial. JAMA Pediatr. 2014;167: 528-536. doi: 10.1001/jamapediatrics.2013.1389

13 Levett-Jones T, Lapkin S. A systematic review of the effectiveness of simulation debriefing in health professional education. Nurse Educ Today. 2014;34: 58-63. doi: 10.1016/j.nedt.2013.09.020

14 Tannenbaum SI, Cerasoli CP. Do team and individual debriefs enhance performance? A meta-analysis. Hum Factors. 2013;55: 231-245. doi: 10.1177/0018720812448394

15 Savoldelli GL, Naik VN, Park J, Joo HS, Chow R, Hamstra SJ. Value of debriefing during simulated crisis management. Anesthesiology. 2006;105: 279-285. doi: 10.1097/00000542-200608000-00010

16 Shinnick MA, Woo M, Horwich TB, Steadman R. Debriefing: the most important component in simulation? Clin Simul Nurs. 2011;7: 105-111. doi:10.1016/j.ecns.2010.11.005

17 Arora S, Ahmed M, Paige J, Nestel D, Runnacles J, Hull L, et al. Objective structured assessment of debriefing: bringing science to the art of debriefing in surgery. Ann Surg. 2012;256: 982-988. doi: 10.1097/SLA.0b013e3182610c91

18 Brett-Fleegler M, Rudolph J, Eppich W, Monuteaux M, Fleegler E, Cheng A, et al. Debriefing assessment for simulation in healthcare: development and psychometric properties. Simul Healthc. 2012;7: 288-294. doi: 10.1097/SIH.0b013e3182620228

19 Imperial College London [Internet]. London: Faculty of Medicine, Imperial College London; c2020 [cited 2020 Jul 20]. The Observational Structured Assessment of Debriefing Tool, Handbook for Debriefing. Available from: https://www.imperial.ac.uk/patient-safety-translational-research-centre/education/training-materials-for-use-in-research-and-clinical-practice/the-observational-structured/

20 American Educational Research Association, American Psychological Association, National Council on Measurement in Education, editors. Standards for educational and psychological testing. Washington, DC: AERA; 2014.

21 Dorer B. Round 6 translation guidelines. Mannheim: European Social Survey, GESIS; 2012.

22 eusim.org [Internet]. EuSim group, c2015 [cited 2020 Jul 20]. Available from: https://eusim.org/

23 Abegglen S, Krieg A, Eigenmann H, Greif R. Rating of debriefings using the Objective Structured Assessment of Debriefing (OSAD) and the German version of the OSAD [dataset]. 2020. Figshare. doi: 10.6084/m9.figshare.13061555

24 Rosseel Y. Lavaan: An R package for structural equation modeling. J Stat Softw. 2012;48(2): 1-36. doi: 10.18637/jss.v048.i02

25 Revelle W. Psych: procedures for personality and psychological research. Version 1.5.4. [software]. 2015 [cited Sept 27 2020]. Available from: http://CRAN.R-project.org/package=psych

26 Pornprasertmanit S, Miller P, Schoemann A, Quick C, Jorgensen T, Pornprasertmanit MS. simsem: SIMulated Structural Equation Modeling. R package version 0.5-15. [software]. 2020 [cited 2020 Sept 27]. Available from: https://CRAN.R-project.org/package=simsem

27 R Core Team. R: a language and environment for statistical computing [software]. R Foundation for Statistical Computing. 2013 [cited 2020 Sept 27]. Available from: http://www.R-project.org/

28 Tong X, Bentler PM. Evaluation of a new mean scaled and moment adjusted test statistic for SEM. Struct Equ Modelling. 2013;20(1): 148-156. doi: 10.1080/10705511.2013.742403

29 Schermelleh-Engel K, Moosbrugger H, Müller H. Evaluating the fit of structural equation models: tests of significance and descriptive goodness-of-fit measures. MPR-online. 2003;8(2): 23-74. doi: 10.1037/1082-989X.9.2.198

30 Bentler PM, Yuan KH. Structural equation modeling with small samples: test statistics. Multivariate Behav Res. 1999;34(2): 181-197. doi: 10.1207/S15327906Mb340203

31 Becker J, Meiring D, Van der Westhuizen JH. Investigating the construct validity of an electronic in-basket exercise using bias-corrected bootstrapping and Monte Carlo re-sampling techniques. SA J Industr Psychol. 2019;45(1): 1-17. doi: 10.4102/sajip.v45i0.1582

32 Dunn TJ, Baguley T, Brunsden V. From alpha to omega: a practical solution to the pervasive problem of internal consistency estimation. Brit J Psychol. 2014;105(3): 399-412. doi: 10.1111/bjop.12046

33 Cicchetti DV. Guidelines, criteria, and rules of thumb for evaluating normed and standardized assessment instruments in psychology. Psychol Assess. 1994;6(4): 284-290. doi: 10.1037/1040-3590.6.4.284

34 Horn JL. A rationale and test for the number of factors in factor analysis. Psychometrika. 1965;30: 179-185. doi: 10.1007/BF02289447

35 Revelle W, Rocklin T. Very simple structure: an alternative procedure for estimating the optimal number of interpretable factors. Multivariate Behav Res. 1979;14(4): 403-414. doi: 10.1207/s15327906mbr1404_2

36 O'Connor BP. SPSS and SAS programs for determining the number of components using parallel analysis and Velicer's MAP test. Behav Res Methods Instr. 2000;32(3): 396-402. doi: 10.3758/BF03200807

37 Fornell C, Larcker DF. Evaluating structural equation models with unobservable variables and measurement error. J Mark Res. 1981;18(1): 39-50. doi: 10.2307/3151312

38 Tavakol M, Dennick R. Making sense of Cronbach's α. Int J Med Educ. 2011;2: 53-55. doi: 10.5116/ijme.4dfb.8dfd

Supporting Information

S1 Appendix. German version of the OSAD (G-OSAD).

S2 Appendix. Typical questionnaire answers for the German version of the OSAD.

Click here to access/download;Figure;Fig 1.tif

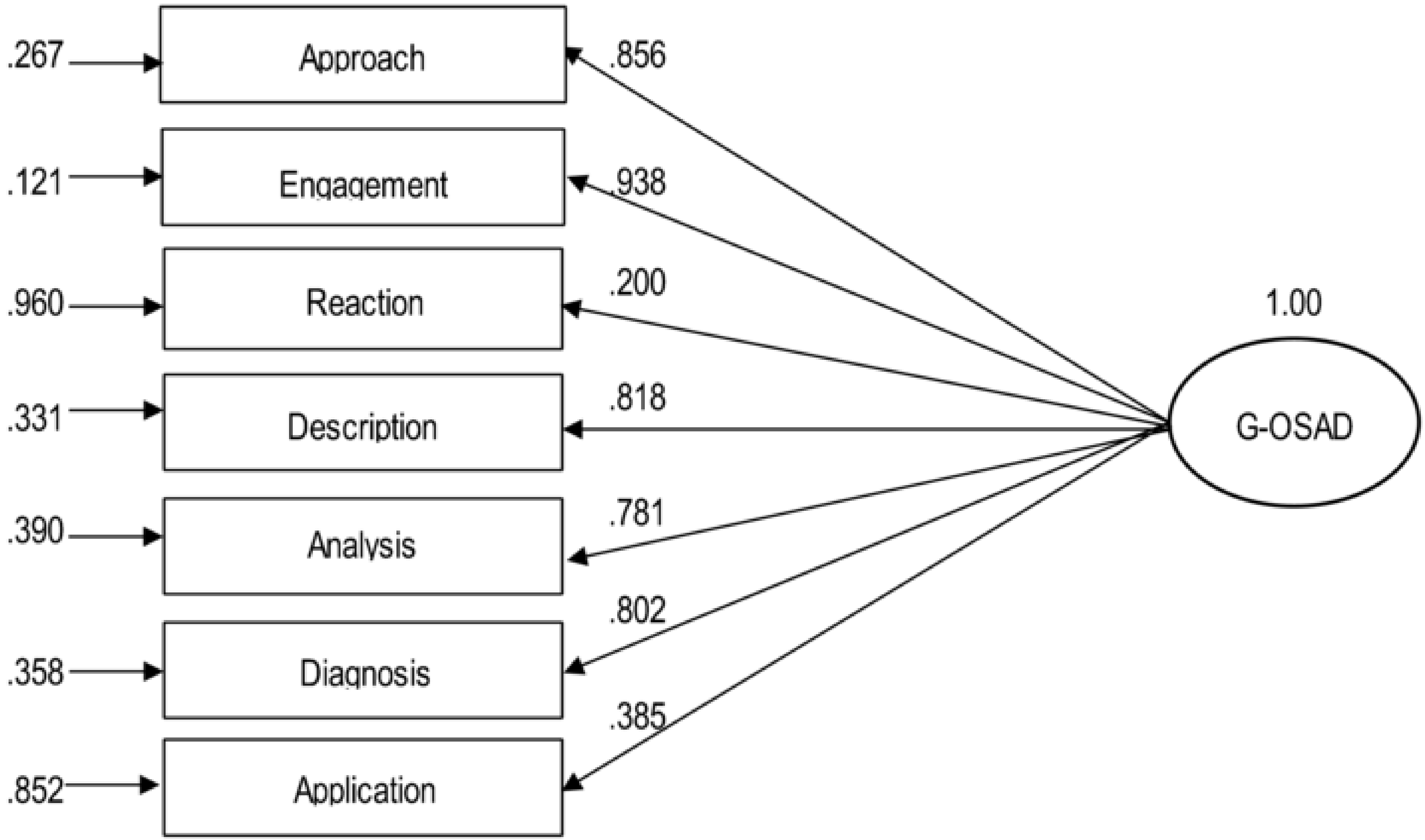

Click here to access/download
Supporting Information
S2_Appendix.pdf

Click here to access/download
Supporting Information
S1_Appendix.pdf

PRIVATE Data Review URL. Only for review during submission. If
the manuscript is accepted for publication, the doi indicated in the

Click here to download Data Review URL
https://figshare.com/s/e723623ef378e3c51c36
