# Supplementary material for: Objective Structured Assessment of Debriefing (OSAD) in simulation-based medical education: Translation and validation of the German version
Source: PLoS One. 2020 Dec 31;15(12):e0244816. doi: 10.1371/journal.pone.0244816 (PMC7774931; doi:10.1371/journal.pone.0244816)
Supplement: Attachment — Submitted filename: Response to Reviewers.docx [file pone.0244816.s004.docx]

Sandra Abegglen, Dr. phil.
University of Bern
Department of Health Psychology and Behavioural Medicine
Fabrikstrasse 6
CH-3012 Bern (Switzerland)
<https://www.gpv.psy.unibe.ch/>
T +41 31 631 54 28
[sandra.abegglen@psy.unibe.ch](mailto:sandra.abegglen@psy.unibe.ch)

16^th^ December, 2020

Dr. Frank Sudzina
Academic Editor

PLOS ONE

Dear Dr. Frank Sudzina,

We submit the revised version of our manuscript **(PONE-D-20-32979)** entitled **“Objective Structured Assessment of Debriefing (OSAD) in simulation-based medical education: Translation and validation of the German version”** for further consideration for publication in the *PLOS ONE.*

We thank you and your reviewers for the valuable comments and suggestions that have helped us to improve our manuscript. We provide here our point-by-point replies to the comments and questions. All changes in the manuscript are highlighted in red.

We have already made our datasets accessible and would like to change our Data Availability statement as follows: “All datasets are available from the figshare data base (https://doi.org/10.6084/m9.figshare.13061555.v1).”

All authors have agreed to the changes and have read and approved the final version of the revised manuscript, as submitted now.

We hope with these changes and adaptation our manuscript is now suitable for publication in *PLOS ONE* and look forward to receiving your reply*.*

Sincerely,


Sandra Abegglen

**Reviewers´ and Editorial comments and our replies:**

**Editor, Frank Sudzina**

Journal Requirements:

When submitting your revision, we need you to address these additional requirements.

*1. Please ensure that your manuscript meets PLOS ONE's style requirements, including those for file naming. The PLOS ONE style templates can be found at*

[*https://journals.plos.org/plosone/s/file?id=wjVg/PLOSOne_formatting_sample_main_body.pdf*](https://journals.plos.org/plosone/s/file?id=wjVg/PLOSOne_formatting_sample_main_body.pdf) *and*

[*https://journals.plos.org/plosone/s/file?id=ba62/PLOSOne_formatting_sample_title_authors_affiliations.pdf*](https://journals.plos.org/plosone/s/file?id=ba62/PLOSOne_formatting_sample_title_authors_affiliations.pdf)

**Our reply:** We already used the templates and ensured that the style requirements were accordingly met.

*2. We note that you have stated that you will provide repository information for your data at acceptance. Should your manuscript be accepted for publication, we will hold it until you provide the relevant accession numbers or DOIs necessary to access your data. If you wish to make changes to your Data Availability statement, please describe these changes in your cover letter and we will update your Data Availability statement to reflect the information you provide.*

**Our reply:** We have already made our datasets accessible and would like to change our Data Availability statement as follows: “All datasets are available from the figshare data base (https://doi.org/10.6084/m9.figshare.13061555.v1).”

We wish to thank the editor and reviewers for their prompt review of our manuscript. We appreciate the comments and suggestions and feel that our manuscript has improved as a result of the peer review process. As requested, we have responded to the reviewers’ comment below. We have added line numbers to our revised manuscript to ease in the review of the changes we have made.

**Reviewer #1, Dr. Marc Lazarovici**

***Comment 1:*** *(P5, L115) What was their experience as SMBE-instructors? I believe in the contecxt of this paper that is rather relevant..*

*(P6, L119) Did they know the original english OSAD-version? This might influence their opinion on the translated version.*

**Our reply:** We thank the reviewer for these valuable questions and the opportunity to clarify the background of the experienced instructors. We have extended the paragraph and have given additional information (Page 5-6, Line 115-119): profession, age, gender, experience, and familiarity with the original OSAD.

***Comment 2:*** *(Page 6, line 134) Please leaobrate on the rationale of choosing three apparently novices in SBME debriefing with no prior healthcare knowledge, which might make understanding and following parts of the debriefings more difficult, as opposed to choosing three experienced SBME-instructors, or at least people with clinical background, for this study. I could imagine this choice having some impact on the rating, as I will discuss further.*

**Our reply:** Thank you for this important comment. We completely agree that understanding the contents of the debriefings might be more difficult for novices in SBME as opposed to SBME instructors/people with clinical background. On the other hand, psychologists are experienced with the rating of behaviour and are specialists for counselling and educational techniques. Further, psychology students are well trained in such ratings of behaviour. In our understanding these skills are the main focus of the OSAD. Thus, we assume that psychology students as experts of these techniques are well prepared for the rating of debriefer behaviours. Moreover, specifically for this study, we (SA & HE) applied an extensive rater training with the students to ensure an adaptation to the specialities of debriefings of simulation-based medical education. This rating training was supervised by a psychologist (SA) which has four years of experience in simulation-based medical education and simulation debriefings. Further, she is a counsellor for the instructors at the Bern University Hospital Simulation Centre. To clarify, we have extended the paragraph (Page 6-7, Line 141 – 143) with this important additional information to clarify this issue. However, if the lack of clinical expertise might have influenced the ratings is an interesting question for future research, specifically if the raters were trained. Thus, we have added information in the discussion section to consider this important limitation (Page 14-15, Line 317-330).

***Comment 3:*** *(Page 7, line 147-150). I consider this to be an interesting weakness in a very sound and well-designed study. Surely, the structure of the SBME course was discussed prior to recording the sessions, so it must have been known that the scene setting was not part of the debriefing. As omitting one out of eight scale elements is not negligeable, I think the reader must get a better understanding on why this was omitted. Were the original recordings of the simulation sessions not available? If so, why not? This really needs further explanations, as the absence of the item is permeating thoughout the subsequent discussion.*

*Do the authors have any plans to revisit this in the future?*

**Our reply:** We thank the reviewer for this question. We wish to clarify this important issue. Our SBME courses last a half day and compromise three different simulation scenarios, and each is followed by a debriefing (facilitated by video recordings of the scenarios). Thus, our instructors establish a learning environment (i.e. clarifying the expectations and objectives from the learner, opportunity to familiarize with the simulation environment) only once - at the beginning of the half day course. At the Bern simulation Centre the introduction is never video recorded. Therefore, this category could not be analysed.

We hope we could clarify the background of why we could not analyse this category in the manuscript. Thus, we have edited this paragraph in the method section (Page 7, Line 152-157) by adding a description of the structure of our courses. Additionally, this limitation of our study is now described in the paragraph of the discussion section (Page 17, 385 - 390). Nevertheless we are convicted that our translation of the category “establishing a learning environment” still will have a practical value for German-speaking debriefers. Therefore, we are planning to further evaluate G-OSAD and to incorporate this category accordingly.

***Comment 4:*** *(Page 9, line 194-195). A more in-depth axplanation on why this was done would be interesting, maybe along with one or two examples. The reason is that maybe some suggestions to deviate from the original scale were due to a different healthcare culture in english versus german-speaking regions. If the suggestions were of a more basic nature, thus altering the core structure of the scale, the decision is perfectly understandable, but I think the reader needs to better understand the process of reaching the decision.*

**Our reply:** Thank you for this thoughtful remark. We have included additional information and examples to the respective paragraph for a more in-depth understanding of our decision (Page 9, Line 201-205). Indeed, adding expert instructors’ suggestions which were of a more basic nature (i.e. adding new behavioural examples in the German version) would endanger the comparability of G-OSAD to the Original OSAD. We hope that this issue is now clearer for the readers.

***Comment 5:*** *(Page 14, line 306, 308, 312). Common comment for the marked sections - maybe there is a third explanation, not considered here: the lack of clinical background of the raters. Maybe a different population of raters might have performed better?*

*Moreover, exactly the two categories "reaction phase" and "application phase" do contain, in my experience, the most clinical references and conversations - thses might have been difficult to follow and to understand for the raters, thus leading to the present results. I think a more in-depth analysis of this aspect might be valuable.*

**Our reply:** Thank you for this important comment and for sharing your appreciated experience. The influence of different rater populations is indeed an important limitation of our study, which we had not sufficiently addressed in the manuscript. We have now revised the marked sections to account for this lack of clarity (Page 14-15, Line 317-318) and (Page 15, Line 324 – 330). Especially, we have pointed out that the lack of clinical experience could be another explanation for a lower interrater reliability of these two categories. On the other hand, all raters were trained psychology students specialized in observing behaviours and counselling. This expertise facilitates the comparability of their ratings and therefore the interrater-reliability itself, as they were all from the same field. Thus, we assume that the low interrater reliability could indicate a general issue of the OSAD. In addition, we have also taken up this point in the limitations (Page 17, Line 381-384). Clearly more research is needed to address this important open question.

***Comment 6:*** *(Page 15, line 326-327:* “The CFA based on the ratings of the 57 debriefings indicated one underlying factor”*). Maybe, to better emphasize the point made, ".. a single underlying factor?"*

**Our reply:** Thank you very much for this suggestion. We have adopted the wording of the underlying factor as you suggested (Page 16, Line 350-351). We feel that the results are now much clearer.

**Reviewer #2**

***Comment 1:*** *You are using the wording: Simulation-based medical education (SBME), what is the difference to simulation-based medical training? And why have you used this phrase?*

**Our reply**: Thank you for pointing out this need for clarification. To our knowledge, “medical training” and “medical education” are often used interchangeably in the literature on simulation in healthcare. Our decision to use “simulation based medical education” is based on a theoretical and a pragmatic argument: First, as we wish to emphasize the benefit of simulation-based approaches to enhance conceptual knowledge, basic skills, and introduction to the actual work. We followed the definition of Gaba (2004, p. i3), who stated: *“The most obvious application of simulation is to improve the education and training of clinicians, but other purposes are also meaningful. Education emphasises conceptual knowledge, basic skills, and an introduction to the actual work. Training emphasises the actual tasks and work to be performed.”*
Second, as we considered strategies for Search Engine Optimization (https://trends.google.com/trends/explore?q=medical%20education,medical%20training), we concluded that the term “medical education” is much more preferred by potential readers and thus raises the visibility of your work and might improve our claims of impact. If the editor has strong concerns regarding the term “medical education” we are willing to incorporate another paragraph on the two different definitions.

***Comment 2:*** *Line 107; (4) Two bilingual speakers with medical backgrounds….
What is the rationale behind 2 independent translators? Not one or several?*

**Our reply:** Thank you for this thoughtful remark. We followed the “Round 6 Translation Guidelines” (Dorer, 2012) of the European Social Survey (ESS) in our validation study to ensure comparability between countries. According to these guidelines there is a need for two independent translators, to enhance the quality of the whole translation and validation process: Dorer (2012, p.9) stated: “*Using one translator may appear to be cheaper and faster than using several translators and would also seem to eliminate the need to co-ordinate teamwork. However, relying on one person to provide a questionnaire translation is problematic, in particular if no team-based assessment is undertaken. A translator working alone and simply ‘handing over’ the finished assignment has no opportunity to discuss and develop alternatives. Regional variance, idiosyncratic interpretations, and inevitable translator blind spots are better handled if several translators are involved and an exchange of versions and views is part of the review process. In addition, survey translations often call for words people say rather than words people write. Group discussion (including input from survey fielding people) is likely to highlight such problems. Team-based approaches include the translators in the review process. Thus the cost of using two translators to translate is offset by their participation in assessment. Since they are familiar with translation problems in the texts, the review is more effective.”*

***Comment 3:*** *Line 127 ……debriefings (duration, 20-59 min.)….. How is the validity of the OSAD in debriefing with significant different timespans?*

**Our reply:** We thank the reviewer for this question. We see this as a general limitation in every validation process. To discuss this limitation, we have extended the discussion section (Page 17, Line 381-384).

***Comment 4:*** *Line 201. Could you please provide more background to the 14 instructors.*

**Our reply:**  Thank you for this important comment. We have extended the paragraph and have given additional information (Page 5-6, Line 115-119): profession, age, gender, experience, and familiarity with the original OSAD.

***Comment 5:*** *Table 1: Could you please provide the possible maximum score for the different G OSAD categories*

**Our reply:** Thank you for pointing out this oversight. We added the following sentence in the notes of table 1 (Page 10, Line 218): “Possible score for the different G-OSAD categories, 1 (minimum) to 5 (maximum)”

***Comment 6:*** *Table 2 The presentation of the different p values should be mor clear. Maybe you can find another table presentation?*

**Our** reply: Thank you for this important remark. We changed the presentation of the *p* values in Table 2 (Page 10-11, Line 231-237) and hope the table is now more readable and helpful for the readers.

***Comment 7:*** *Line 270 Could you please discuss and present the results of the convergent validity in the limitations?*

**Our reply:** Thank you for this question. To clarify this point we have described and discussed this important aspect of validity in the discussion section as suggested (Page 16, Line 354-361).

***Comment 8:*** *Line 315; why have you chosen a short timespan for the examination of the test-retest reliability?*

**Our reply:** Thank you for this important question. We agree that it should be noted that the determined correlation may vary depending on the time interval between the two ratings (De Vet, Terwee, Mokkink, & Knol, 2011). Compared to the study on the original English OSAD we chose a shorter time span, because we strived to a reasonable compromise between recollection bias and unsystematic changes in perception and behaviour of the trained student raters (i.e. uncontrolled knowledge growth due to different lectures, variations in performance due to different exam burden) (De Vet, Terwee, Mokkink, & Knol, 2011). To clarify this critical point, we have extended our discussion section with the respective reference to De Vet and collegues (2011) and discuss the time span as a critical point (Page 15, Line 334-339). Nevertheless, we are convicted that the ratings of 57debriefings compared to translation of the category “establishing a learning environment” still will have a practical value for German-speaking debriefers.
